# Генетический профиль образований околощитовидных желез: приоткрывая завесу тайны

**DOI:** 10.14341/probl13543

**Published:** 2025-05-20

**Authors:** Х. В. Багирова, О. Ю. Спасская, Е. И. Ким, А. А. Лавренюк, А. К. Еремкина, Н. Г. Мокрышева

**Affiliations:** Национальный медицинский исследовательский центр эндокринологии; Национальный медицинский исследовательский центр эндокринологии; Национальный медицинский исследовательский центр эндокринологии; Национальный медицинский исследовательский центр эндокринологии; Национальный медицинский исследовательский центр эндокринологии; Национальный медицинский исследовательский центр эндокринологии

**Keywords:** первичный гиперпаратиреоз, образования околощитовидных желез, генетические методы исследования, сравнительная геномная гибридизация, полноэкзомное секвенирование, полногеномное секвенирование, РНК-секвенирование, эпигенетика

## Abstract

Первичный гиперпаратиреоз (ПГПТ) — частое эндокринное нарушение, характеризующееся автономной секрецией паратгормона измененными околощитовидными железами (ОЩЖ). В большинстве случаев ПГПТ является спорадическим заболеванием, 5–10% наблюдений приходится на долю генетически детерминированных синдромальных и несиндромальных форм. Изучение семей с наследственными формами ПГПТ привело к открытию основных генов-онкосупрессоров и протоноонкогенов, соматические мутации которых лежат в основе развития многих спорадических опухолей ОЩЖ. Отдельный интерес в контексте патогенеза первичного гиперпаратиреоза уделяется изучению механизмов эпигенетической регуляции в ткани опухоли. В первой части обзора будут рассмотрены вопросы классификации, морфологии и этиологии ПГПТ. Во второй мы представим резюме основных исследований с применением генетического анализа, разделив их в зависимости от применяемого метода.

## Abstract (en)

Primary hyperparathyroidism (PHPT) is a common endocrine disorder characterized by autonomous secretion of parathyroid hormone by altered parathyroid glands. In most cases PHPT is a sporadic disease, 5-10% of observations are genetically determined syndromal and non-syndromal forms. Studies of families with hereditary forms of PHPT have led to the discovery of key oncosuppressor genes and proto-oncogenes whose somatic mutations underlie the development of many sporadic parathyroid tumors. Another interest in the pathogenesis of primary hyperparathyroidism is studying mechanisms of epigenetic regulation in tumor tissue. In the first part of this review, we will discuss the classification, morphology, and etiology of PHPT. In the second part, we will present a summary of the most important studies using genetic analysis, classified according to the method used.

## ВВЕДЕНИЕ

Первичный гиперпаратиреоз (ПГПТ) — частое эндокринное заболевание с распространенностью 0,4–82 случая на 100 000 человек [1]. ПГПТ характеризуется автономной секрецией паратиреоидного гормона (ПТГ) измененными околощитовидными железами (ОЩЖ), а также верхне-нормальным или повышенным уровнем кальция крови [2]. В большинстве случаев ПГПТ является спорадическим заболеванием, 5–10% наблюдений приходится на долю наследственных синдромальных и несиндромальных форм [3][4]. Как правило, ПГПТ диагностируется на 5–6 декадах жизни, однако для наследственных форм характерна более ранняя манифестация заболевания [5].

Клиническая картина ПГПТ варьирует от неспецифических симптомов, таких как слабость, повышенная утомляемость, снижение эмоционального фона, до ярких проявлений, связанных с поражением ключевых «органов-мишеней». К ним относится нефролитиаз/нефрокальциноз, язвенная болезнь желудка и двенадцатиперстной кишки, генерализованные боли и деформация скелета, остеопороз, кистозно-фиброзный остеит [6]. Вопрос о «неклассических» проявлениях ПГПТ остается актуальным, в ряде исследований отмечена ассоциация ПГПТ с развитием патологии сердечно-сосудистой системы и когнитивными нарушениями [7].

В настоящее время основным методом лечения ПГПТ остается хирургический. Объем операции зависит от количества пораженных ОЩЖ, злокачественного потенциала опухоли. Для большинства пациентов с ПГПТ и солитарной аденомой операцией выбора является селективная паратиреоидэктомия (ПТЭ). При мультигландулярном поражении чаще проводится субтотальная или тотальная ПТЭ. В случае карциномы ОЩЖ оптимальным объемом считается удаление опухоли с ипсилатеральной долей ЩЖ (резекция en bloc). Однако методы топической диагностики при множественном поражении имеют достаточно низкую чувствительность и специфичность и не всегда позволяют достоверно визуализировать все измененные ОЩЖ на дооперационном этапе [8][9], надежные критерии предоперационной диагностики карциномы отсутствуют [10][11], эти факторы сопряжены с высоким риском персистенции/рецидива при мультигландулярном и злокачественном поражении ОЩЖ. Таким образом, существует потребность в изучении молекулярных механизмов патогенеза и поиске специфических биомаркеров, на основании которых можно планировать оптимальную тактику в каждом конкретном случае. Кроме того, анализ молекулярно-биологического профиля карциномы ОЩЖ может способствовать выявлению потенциальных мишеней для таргетной терапии.

В первой части обзора будут рассмотрены вопросы классификации, морфологии и этиологии ПГПТ, как при спорадических, так и наследственных формах. Во второй мы представим резюме основных исследований с применением генетического анализа, разделив их в зависимости от применяемого метода.

## КЛАССИФИКАЦИЯ ОПУХОЛЕЙ ОКОЛОЩИТОВИДНЫХ ЖЕЛЕЗ

Классификация опухолей эндокринных органов Всемирной организации здравоохранения (ВОЗ) 2022 г. (5-е издание) включает 4 основных типа образований ОЩЖ: аденома, атипическая опухоль (АО), карцинома и множественные неоплазии [12]. Эксперты ВОЗ предложили отойти от ранее используемого термина «гиперплазия» в контексте ПГПТ и оставить его только для вторичного гиперпаратиреоза вследствие хронической болезни почек (ХБП). Данные изменения в классификации аргументированы тем, что при полигландулярном поражении у пациентов с ПГПТ наблюдаются множественные «клональные» неопластические процессы, поэтому более правильным является термин «множественные аденомы/множественные неоплазии ОЩЖ». Стоит также отметить, что в новом издании «атипическая аденома» была заменена на «атипическую опухоль», что подчеркивает ее неопределенный злокачественный потенциал [13]. Новая классификация ВОЗ 2022 г. также вводит понятие опухолей ОЩЖ со сниженной экспрессией парафибромина, тем самым подчеркивая необходимость проведение молекулярно-генетического исследования для выявления герминальной мутации CDC73.

ПГПТ в 85–90% случаев обусловлен солитарной аденомой ОЩЖ, примерно в 5–10% случаев — множественными неоплазиями ОЩЖ; в 1% — раком ОЩЖ. Частота АО ОЩЖ варьирует от 0,5 до 4,4% случаев, однако истинная распространенность неизвестна.

Солитарные аденомы чаще встречаются у женщин, чем у мужчин, в соотношении 3:1 [14]. Большинство из них — инкапсулированные новообразования, состоящие из главных клеток, находящихся в разных стадиях секреторного цикла. До 20% аденом могут быть эктопированы, в основном в средостение, щитовидную железу, трахеопищеводную борозду и ретроэзофагеальные ткани.

При АО соотношение женщин и мужчин составляет 1,5:1 [15]. Диагноз устанавливается в ходе морфологического исследования при наличии подозрительных в отношении злокачественного потенциала признаков: фиброзные тяжи, некрозы, высокая митотическая активность (>5/50 полей зрения при большом увеличении), ядерная атипия, солидный или трабекулярный тип строения, сращение с соседними структурами (без прорастания), наличие опухолевых клеток в окружающей капсуле, но при отсутствии достоверных признаков инвазии. В случае возникновения регионарных или отдаленных метастазов за время наблюдения диагноз АО может быть пересмотрен [16].

Карцинома ОЩЖ встречается в равной степени у мужчин и женщин [17]. Морфологические критерии карциномы включают истинные признаки инвазивного роста (сосудистую и/или лимфатическую, и/или периневральную, и/или инвазию в соседние структуры), а также наличие документированных метастазов [12].

Множественное (полигландулярное) поражение подразумевает вовлечение в патологический процесс синхронно или метахронно двух и более ОЩЖ, может развиваться как в рамках наследственных синдромов, и быть спорадическим. По данным литературы, частота множественного поражения при спорадическом ПГПТ варьирует от 8 до 33% [18][19]. Насколько морфологическая картина при наследственных формах ПГПТ сопоставима со спорадическим первично множественным поражением ОЩЖ, остается неясным [12][20].

## ЭТИОЛОГИЯ ПГПТ

Ранее пожилой возраст, женский пол, дефицит эстрогенов, облучение шеи в анамнезе рассматривались в качестве ключевых факторов развития ПГПТ, но на сегодняшний день их главенствующая роль представляется сомнительной [21]. Развитие генетических методов исследования стало новым шагом в понимании туморогенеза ОЩЖ.

В настоящее время выделяют 2 ключевых гена, ассоциированных с развитием спорадических аденом ОЩЖ при ПГПТ, — MEN1 и CCND1, что также было подтверждено в экспериментальных работах на мышиных моделях. Оба гена расположены на 11-й хромосоме (11q13), как и ген, кодирующий синтез паратиреоидного гормона (PTH, 11p15) [22][23].

Биаллельные инактивирующие соматические мутации в MEN1 наблюдаются в 25–40% случаев спорадических опухолей ОЩЖ, что является наиболее частым генетическим соматическим нарушением [24][25]. Интересно, что началом поиска соматических мутаций в этом гене при спорадических новообразованиях послужила первоначальная его идентификация в 1997 году с помощью позиционного клонирования у членов семьи с синдромом МЭН-1 [26].

Менин, продукт гена MEN1, представляет собой повсеместно экспрессируемый, преимущественно ядерный белок с молекулярной массой 67 кДа, без собственной ферментативной активности. Ранее менин рассматривался только в качестве опухолевого супрессора, в настоящее время воспринимается скорее как молекулярный адаптер. С помощью рецепторного «кармана» менин связывается и с транскрипционным фактором JunD (относящийся к семейству белка-активатора 1 и участвующий в подавлении пролиферативной активности клетки), и с гистон-модифицирующей метилтрансферазой (MLL1), однако эффекты от этих взаимодействий диаметрально противоположны. В первом случае запускается онкосупрессия, во втором — наоборот рост опухоли [27].

Перицентрические инверсии 11-й хромосомы с участием промотора генов PTH и CCND1 обнаруживаются примерно в 8% спорадических образований ОЩЖ, при этом гиперэкспрессия CCND1 наблюдается значимо чаще — в 20–40% аденом и примерно в 90% карцином ОЩЖ [28]. Циклин D1, кодируемый CCND1, регулирует переход G1/S в клеточном цикле. Циклин D1 способствует фосфорилированию белка ретинобластомы (Rb) и других субстратов путем связывания с циклин-зависимой киназой 4/6 (CDK4/6 — cyclin-dependent kinase 4/6), что приводит к быстрому делению клеток. Инверсия 11 хромосомы вблизи центромеры ассоциирована со смещением промоторной последовательности гена PTH непосредственно перед CCND1, что приводит к усилению экспрессии циклина D1 и активации CDK [29].

В патогенезе спорадических аденом изучается роль генов, кодирующих ингибиторы циклин-зависимых киназ (CDKN1A, CDKN1B, CDKN1C, CDKN2A, CDKN2C, CDKN2). Ингибиторы циклин-зависимой киназы — белки, блокирующие активность CDK отдельно или в комплексе с циклином в фазе G1 клеточного цикла. Также описаны мутации в генах кальций чувствительного рецептора (CASR), бета-катенина (CTNNB1), метилтрансферазы, катализирующей триметилирование гистона НЗ по лизину 27 (EZH2), Х-связанного белка цинкового пальца (ZFX). Однако их вклад в развитие заболевания все еще неопределенный [30].

Карцинома ОЩЖ в большей степени ассоциирована с мутациями в гене СDC73, расположенном на длинном плече 1-й хромосомы (1q31.2). Он состоит из 17 экзонов и кодирует белок парафибромин. Более 75% спорадических карцином ОЩЖ имеют двуаллельную соматическую инактивацию/потерю гена CDC73, а отсутствие экспрессии парафибромина наблюдается в 33–62% случаев. Парафибромин — компонент полимераза-ассоциированного фактора 1 (PAF1), взаимодействующего с РНК-полимеразой II типа в процессе транскрипции ДНК. В зависимости от типа клетки парафибромин может функционировать как онкосупрессор или онкоген. В опухолях ОЩЖ потеря гетерозиготности в локусе 1q31.2 у пациентов с герминальными мутациями гена CDC73 указывает на биаллельную инактивацию гена, что подтверждает роль парафибромина как онкосупрессора. Соматические мутации в гене CDC73 в спорадических аденомах могут выявляться в 4% случаев [31][32].

Работы, посвященные генетическому профилированию при АО ОЩЖ, лимитированы. В отличие от аденом и карцином, при АО мутации в генах MEN1 и CDC73 определяются редко [15]. Имеются данные о потенциальной роли генов CDKN1A, CDKN2A, RB, а также гена фермента липидной фосфатазы PTEN. Ген PTEN кодирует фосфатазу с двойной субстратной специфичностью, негативно регулирующей PI3K/AKT/mTOR-сигнальный путь, что делает ее онкосупрессором [33].

Этиология генетически детерминированных форм ПГПТ представляет собой более изученный вопрос. Несмотря на идентифицированные мутации, «избирательность» поражения органов эндокринной системы до сих пор не имеет четкого молекулярно-генетического обоснования. При наследственных синдромах герминальная мутация в генах-онкосупрессорах сопровождается соматической мутацией в ткани опухоли, что и приводит к потере гетерозиготности. Эта генетическая модель неоплазии, включающая две рецессивные мутации в развитии опухолей, известна как гипотеза двух ударов Альфреда Кнудсона [34][35]. Основные наследственные формы ПГПТ и их генетические причины представлены в таблице 1.

Т

**Table:** аблица 1. Наследственные синдромальные и несиндромальные формы ПГПТ Сокращения: МЭН1 — синдром множественных эндокринных неоплазий 1 типа; МЭН2А — синдром множественных эндокринных неоплазий 2А типа; МЭН4 — синдром множественных эндокринных неоплазий 4 типа; HPT-JT — синдром гиперпаратиреоза с опухолью челюсти; NSPHT — неонатальный тяжелый гиперпаратиреоз; FHH — семейная гипокальциурическая гиперкальциемия; FIHP — семейный изолированный гиперпаратиреоз.

Заболевание	Генетическая причина	Тип наследования	Основные клинические проявления
МЭН1	MEN1 (продукт — менин, преимущественно онкосупрессор)	Аутосомно-доминантный	ПГПТ Опухоли аденогипофиза Нейроэндокринные опухоли поджелудочной железы
МЭН2А	RET (продукт — рецепторная тирозинкиназа, протоонкоген)	Аутосомно-доминантный	Медуллярный рак щитовидной железы Феохромоцитома ПГПТ
МЭН4	СDNK1B (продукт — ингибитор циклин-зависимой киназы 1B, p27, онкосупрессор)	Аутосомно-доминантный	ПГПТ Опухоли аденогипофиза, Нейроэндокринные опухоли ЖКТ и легких Образования надпочечников
HPT-JT	CDC73 (продукт — парафибромин, преимущественно онкосупрессор)	Аутосомно-доминантный	ПГПТ Оссифицирующие фибромы нижней челюсти Опухоли почек и матки
NSPHT	CASR (продукт — белок кальций-чувствительного рецептора) Инактивирующие мутации	Аутосомно-рецессивный или аутосомно-доминантный	ПГПТ с рождения или в течение шести первых месяцев жизни
FHH	1 тип: СASR (инактивирующие мутации) 2 тип: GNA11 (продукт — альфа-субъединица гетеротримерного белка Gα11 3 тип: AP2S1 (продукт — белок-адаптер 2 σ-субъединицы)	Аутосомно-доминантный	CCCR<0,01 Гипокальциурия Повышение ПТГ в 20% случаев
FIHP	CASR, MEN1, или CDC73 в 30% случаев Ген GCM2 — в 20% семей с FIHP (продукт — транскрипционный фактор, участвующий в развитии ОЩЖ)	Различные варианты наследования	Изолированный ПГПТ

## СРАВНИТЕЛЬНАЯ ГЕНОМНАЯ ГИБРИДИЗАЦИЯ

Возможность анализировать хромосомные аберрации появилась благодаря методу сравнительной геномной гибридизации (CGH), опыт применения которого для исследования ткани опухолей впервые был описан Kallioniemi A. и соавт. [36]. CGH оказалась мощным инструментом в изучении генетических механизмов развития опухолей ОЩЖ на уровне хромосомных изменений, а также основой для дальнейших исследований с применением передовых геномных технологий.

В 1998 г., Agarwal S.K. и соавт. провели сравнительный анализ 10 аденом и 10 карцином ОЩЖ с использованием CGH. При аденомах хромосомные аберрации наблюдались практически во всех хромосомах, за исключением 8, в то время как в карциномах хромосомы 2, 9, 10 и 21 не претерпевали никаких изменений. В аденомах чаще наблюдались делеции 11, 17 и 22-й хромосомы, а в карциномах — делеции 1р и 17-й, а также дупликации 5-й хромосомы. В 40% случаев карцином была выявлена потеря аллеля на хромосомном плече 1p. В 2/10 случаев аденом наблюдалась полная утрата 11-й хромосомы, а в двух других — утрата ее длинного плеча 11q [37].

Позднее, в 1999 г., Farnebo F. и соавт. провели CGH анализ 44 солитарных аденом ОЩЖ. Было установлено, что в спорадических аденомах делеции чаще происходят в хромосомах 11 (38%), 15q (27%) и 1p (19%), в то время как амплификации преимущественно выявляются в 19p (15%) и 7p (12%). Также были обнаружены множественные аберрации в спорадических опухолях с соматической мутацией и/или потерей гетерозиготности MEN1 [38].

Kytölä S. и соавт. проанализировали 29 образцов карцином ОЩЖ. Хромосомные аберрации определялись в 86% опухолей, при этом дупликации и потери/делеции обнаруживались с одинаковой частотой. Чаще всего были выявлены потери 1p и 13q — более чем в 40% случаев. При сравнении с ранее опубликованными данными было установлено, что для карцином ОЩЖ в отличие от доброкачественных образований более характерна потеря 1p, 4q и 13q, а также дупликация 1q, 9q, 16p, 19p и Xc. Потеря локуса 11q13, была частой в спорадических аденомах и не была обнаружена в карциномах [39], что согласуется с вышеописанными работами.

Dwight T. и соавт. применили CGH для изучения мультигландулярного поражения ОЩЖ [40]. Изучено 10 образований ОЩЖ, полученных от пяти пациентов, прооперированных по поводу спорадического ПГПТ (установленного клинически, генетического анализа не проводилось). Наиболее частыми изменениями были потери 11 (22%) и 13q (22%) хромосом. Ни в одной из парных опухолей, полученных от одного и того же пациента, не было обнаружено одинаковых изменений числа копий последовательности ДНК. В 30% опухолей с потерей гетерозиготности в 11q13, наблюдалась соматическая мутация гена MEN1. Интересно, что мутация выявлялась только в одной из парных опухолей пациента.

## Полноэкзомное секвенирование

Более глубокое изучение молекулярных механизмов развития опухолей ОЩЖ стало возможным благодаря технологиям высокопроизводительного секвенирования (next-generation sequencing, NGS), таким как полноэкзомное секвенирование (whole exome sequencing, WES) и секвенирование тотальной РНК (bulk RNA sequencing, RNA-seq). Именно с помощью высокопроизводительного секвенирования удалось обнаружить новые предполагаемые драйверные гены, включая гены EZH2, ZFX, CTNNB1, CDKN1B в случае аденом, и PIK3CA/MTOR, ADCK1, PRUNE2, ZEB1 для карцином.

В исследовании Newey P.J. и соавт. 2012 г. представлен анализ экзома 16 аденом ОЩЖ [41]. Авторы исключили герминальные мутации в генах MEN1, CDC73, RET, CDKN1B и CASR, ассоциированных с наследственными случаями ПГПТ. В ткани опухолей суммарно было идентифицировано 255 опухолеспецифических (соматических) мутаций, из них 212 подтверждены дидезоксинуклеотидным секвенированием. Из 212 вариантов однонуклеотидные замены составили 93% (n=197), а инсерции или делеции — 7% (n=15). Большая часть таких вариантов (52%) обнаружена в опухоли #6, полученной от пациента с клинически «агрессивным» ПГПТ. Хотя при гистологическом исследовании опухоль не демонстрировала признаков атипии. Этот образец анализировался отдельно. Была выявлена новая мутация c.G546C в гене POT1, являющегося ключевым регулятором целостности теломер и стабильности генома. Интересно, что потеря гетерозиготности, затрагивающая 11 хромосому, где расположен ген MEN1, наблюдалась более чем в 45% (7/15) опухолей, среди них в 85% (6/7) определялась также соматическая мутация гена MEN1. В то же время все опухоли, несущие соматические мутации MEN1, имели дополнительные соматические мутации (~7,6 дополнительных мутаций на опухоль) в генах, которые вовлечены в туморогенез, контроль клеточного цикла и регуляцию стабильности генома. Возможно, для развития опухоли недостаточно лишь соматической мутации MEN1, что соответствует многоступенчатой парадигме опухолевого генеза.

Наиболее крупное исследование с применением WES проведено в китайской популяции: проанализировано 73 аденомы ОЩЖ. Наиболее часто выявлялись соматические мутации гена KMT2D (15/73 образцов). Ген KMT2D расположен на 12q13.12 и содержит 56 экзонов. Продукт гена — специфическая гистон-метилтрансфераза 2D, метилирует остаток лизина в положении 4 гистона H3, необходима для эмбриогенеза и функционирует как усилитель экспрессии других генов [42]. У 5 пациентов с аденомами ОЩЖ выявлены соматические мутации в гене CDC73. Данные пациенты характеризовались более высокими дооперационными значениями ПТГ и кальция, более молодым возрастом, а также сниженной экспрессией парафибромина по данным иммуногистохимического анализа [43].

Yu W. и соавт. провели сравнительный анализ 9 образцов рака и 40 аденом ОЩЖ [44]. Были обнаружены как герминальные, так и соматические мутации гена CDC73 в 7/9 образцов карцином ОЩЖ. В двух образцах опухолей была обнаружена только гетерозиготная мутация гена CDC73, помимо которой были выявлены только 3–4 синонимичные соматические мутации. В 4 случаях выявлено около 3–5 копий мутантных аллелей CDC73, в 3 образцах — потеря аллеля дикого типа за счет делеций или потери плеча хромосомы. Интересно, что феномен усиления мутированных аллелей ранее также был описан при папиллярном раке почки для протоонкогена Met [45]. В 18% случаев карцином были выявлены мутации гена PRUNE2, в том числе герминальная миссенс-мутация у пациента с диким типом CDC73. PRUNE2 расположен на 9-й хромосоме и кодирует белок, регулирующий дифференцировку и выживаемость клеток путем подавления активности RhoA киназы. Сообщалось, что PRUNE2 функционирует как ген-онкосупрессор при раке предстательной железы.

Исследование было продолжено Panday C. и соавт., которые дополнили его полноэкзомным секвенированием еще 10 образцов рака ОЩЖ (суммарно 17 образцов) [46]. В 47% (8/17) опухолей определялись соматические мутации в гене CDC73, при этом у 4/8 пациентов были обнаружены герминальные варианты. Амплификация гена CCND1 выявлена в 29% наблюдений. Впервые описана повторяющаяся замена аминокислоты p.Ile482Met в гене ADCK1, кодирующем белок AarF Domain-Containing Kinase 1 (ADCK1) в 2/17 случаев (11,8%). Белок, кодируемый ADCK1, преимущественно расположен во внутренней мембране митохондрий и необходим для адекватной работы органеллы. Мутации ADCK1 были описаны при раке толстого кишечника, в качестве основного механизма рассматривается активация пути Wnt/β-катенина. Также была выявлена повторяющаяся мутация в гене AKAP9 (17,6%), кодирующем белок семейства белков-якорей протеинкиназы А. В трех случаях были обнаружены две замены аминокислот и одна нонсенс-мутация, приводящая к биаллельной инактивации гена, что указывает на онкосупрессивную функцию гена AKAP9 в ОЩЖ. В ряде образцов выявлены соматические мутации в известных онкогенах, таких как PIK3CA (3 образца) и MTOR (2 образца). Другие исследования также выдвигают предположения о роли пути PIK3CA/AKT/mTOR в канцерогенезе при спорадическом раке ОЩЖ [47].

## Полногеномное секвенирование

Hu Y. и соавт. использовали полногеномное секвенирование (whole-genome sequencing, WGS) для анализа образцов ткани рака ОЩЖ, полученных от 23 пациентов [48]. Потеря PIK3CA была обнаружена в 1 образце, который также имел мутацию в CDC73. При этом мутации генов пути PI3K/AKT/mTOR были обнаружены в 78,3% (18/23) опухолей. Опухолевая мутационная нагрузка оказалась выше у пациентов с раком ОЩЖ и мутацией CDC73 в сравнении с пациентами с CDC73 дикого типа (1,64 против 0,69 на миллион, P=0,026) и была ассоциирована с высоким риском рецидива. Помимо мутаций в гене CDC73 (50%), также определялись мутации в генах филаггрина 2 — FLG2 (21,4%) и гистосовместимости HLA-A (21,4%), и HLA-B. Количество вариантов этих соматических мутаций не коррелировало с рецидивом/метастазированием.

Интересные результаты были получены Kasaian K. и соавт., которые с помощью метода WGS изучили первичную карциному ОЩЖ, а также вторичные очаги при последующих рецидивах [49]. Такое молекулярное профилирование позволило выявить однонуклеотидные точечные мутации в генах mTOR, MLL2, CDKN2C и PIK3CA. Сравнение выявленных мутаций в первичных и рецидивных опухолях выявило потерю активирующей мутации PIK3CA при прогрессии опухоли, это может указывать на то, что PIK3CA играет роль в запуске онкогенеза, но не в его поддержании. PIK3CA кодирует каталитическую субъединицу p110α PI3K, липидную киназу, играющую важную роль в сигнальных путях и, следовательно, в регуляции роста и пролиферации клеток [50]. Потеря этой мутации из доминирующего клона при рецидиве подчеркивает необходимость мониторинга опухоли на молекулярном уровне, поскольку изменения в ее мутационном профиле могут влиять на возможности таргетной терапии [49].

## РНК-секвенирование

Транскриптомное профилирование является основой трансляционных исследований рака и все чаще находит применение в фундаментальной и клинической медицине, в том числе оценке потенциальной чувствительности опухоли к лекарственным препаратам [51]. Однако стоит отметить ряд ограничений: в первую очередь, сложность выявления межклеточных взаимодействий между опухолевыми клетками и клетками иммунной системы. При транскриптомных исследованиях также поступает информация от клеток микроокружения, что осложняет выявление истинных причин резистентности. Развитие методов, нацеленных на секвенирование генетического материала единичных клеток, может преодолевать данные ограничения, так как исключает масштабную примесь клеток микроокружения.

В 2023 г. исследователи из Кореи Jo S.Y. и соавт. проанализировали транскриптом 28 аденом, 11 карцином и 10 неизмененных ОЩЖ [52]. Мутации в гене CDC73 были ассоциированы со сверхэкспрессией WT1 (опухолевого белка Вильямса), что подтверждалось как на уровне РНК, так и на уровне самого пептида (при ИГХ окрашивании). Таким образом, авторы выделили его как перспективный биомаркер карцином ОЩЖ с мутацией CDC73. WT1 — белковый продукт одноименного гена-онкосупрессора, локализованного в 11-й хромосоме, участвующий в развитии тканей, происходящих из мезодермы. Дополнительно было показано, что в 36% случаев карцином (4/11) отмечаются мутации зародышевой линии в гене OGDHL, кодирующем изофермент 2-оксоглутаратдегидрогеназа-подобного белка. Данный белок участвует в цикле трикарбоновых кислот — ключевом этапе дыхания всех клеток. Считается, что данный ген ингибирует рост и миграцию клеток за счет подавления сигнала протеинкиназы в пути PI3K/AKT/mTOR. Также при исследовании китайской популяции было показано, что некоторые варианты мутаций в OGDHL значимо коррелируют с раком молочной железы [53]. Помимо этого, продемонстрировано повышение экспрессии продуктов генов ангиопоэтин-подобного белка 4 (ANGPTL4), трансформирующего кислотного спиральсодержащего белка 3 (TACC3) и тромбоспондина 1 (THBS1). Результаты экспериментальных работ предполагают взаимосвязь между активацией данных генов и инвазией/миграцией опухолевых клеток: ANGPTL4 путем регулирования сосудистой проницаемости и стимуляции ангиогенеза [54], TACC3 — за счет активации перехода G1/S и пути Wnt [55], а THBS1 — через взаимодействие с поверхностным клеточным гликопротеином CD47, препятствующим фагоцитозу опухолевых клеток [56].

Haven C.J. и соавт. на основании анализа профиля экспрессии РНК в 53 образцах опухолей ОЩЖ выяснили, что гистон H1, белок-предшественник бета-амилоида и E-кадгерин также могут быть использованы как маркеры карцином ОЩЖ. Наличие герминальной или соматической мутации CDC73 оказывает сильное влияние на паттерн экспрессии этих белков [57]. Гистон H1 взаимодействует с линкерной ДНК между нуклеосомами и участвует в уплотнении хроматина в структуры более высокого порядка. Также белок связан с подавлением экспрессии генов и часто сверхэкспрессируется при злокачественных опухолевых процессах [58].

Forsberg L. и соавт. для формирования представления о генетической этиологии развития опухолей ОЩЖ использовали анализ микрочипов и ПЦР в реальном времени для сравнения экспрессии генов в аденомах (n=8) и в здоровых тканях (n=2): была отмечена сверхэкспрессия таких предполагаемых онкогенов, как ген CCND1 и протоонкоген c-Jun, участвующих в регуляции клеточного цикла и матричных процессах [59].

Ученые из Кореи провели сравнительный транскриптомный анализ аденом (n=10) и неизмененных ОЩЖ (n=5). В совокупности было выделено 247 генов, экспрессирующихся при аденомах, некоторые из них, в частности MED12, KMT5A, BMP2K и ATAD2 могут играть важную роль в туморогенезе. Так, белок MED12, кодируемый геном MED12, необходим для активации киназы CDK8, регулирующей процессы транскрипции. Продукт гена KMT5A представляет собой лизин-специфическую N-метилтрансферазу, которая монометилирует лизин 20 в гистоне H4. Известно, что метилирование гистоновых лизинов может быть ассоциировано как с активацией, так и с подавлением экспрессии генов, в зависимости от типа гистона и положения модифицированного аминокислотного остатка в нем [60]. Повышение экспрессии гена KMT5A описано также при папиллярном раке щитовидной железы, раке поджелудочной железы, раке мочевого пузыря, немелкоклеточной и мелкоклеточной карциноме легких, при хроническом миелоидном лейкозе, гепатоцеллюлярной карциноме. BMP2K и ATAD2 участвуют в клеточной пролиферации и регуляции транскрипции, что позволяет предположить их потенциальный вклад в онкогенез [61].

## Эпигенетические факторы

В последнее время большее внимание уделяется роли эпигенетических факторов, а именно метилированию ДНК, модификации гистонов и нарушению регуляции микроРНК, которые влияют на активность гена без изменений в его кодирующей последовательности. Метилирование ДНК заключается в ковалентном присоединении метильной группы к цитозину в 5’-положении, за которым обычно следует гуанин (динуклеотид CpG). Большая часть CpG-динуклеотидов распределена по геному в виде одиночных динуклеотидов, оставшаяся формирует зоны скопления CpG — CpG-островки, присутствующие в промоторных областях 70% генов. Гиперметилирование CpG-островков является обратимым механизмом сайленсинга — тканеспецифичного подавления экспрессии генов [32][62]. В отличие от большинства опухолей, где основным эпигенетическим механизмом туморогенеза выступает гипометилирование протоонкогенов, образования ОЩЖ характеризуются преимущественно гиперметилированием генов-онкосупрессоров, таких как APC, CTNNB1, RASSF1A и SFRP1 [63]. Starker и соавт. выявили гиперметилирование генов — регуляторов клеточного цикла и транскрипции (CDKN2B, CDKN2A, WT1, RASSF1A и PRDM2), а также компонентов сигнального пути Wnt/β-катенин (APC, SFRP1, SFRP2 и SFRP4) [64].

Накопление β-катенина в клетках вследствие дисрегуляции WNT сигнального пути играет одну из ключевых ролей в развитии злокачественных образований желудка, поджелудочной железы, печени, толстой кишки, эндометрия, а также опухолях ОЩЖ у пациентов с ПГПТ и вторичным гиперпаратиреозом [65][66][67][68][69].

Основные эпигенетические механизмы туморогенеза опухолей ОЩЖ представлены в таблице 2.

**Table:** Таблица 2. Гиперметилированные гены опухолей ОЩЖ и их роль в туморогенезе

Ген	Функция кодируемого белка	Молекулярные и клеточные эффекты гиперметилирования промотора
RASSF1A[70]	Онкосупрессор, регулирующий клеточную пролиферацию и апоптоз	Cайленсинг гена RASSF1A приводит к накоплению активной формы β-катенина и к последующей активации канонической передачи сигналов Wnt, что запускает транскрипцию TCF/LEF-чувствительных генов-мишеней, включая CCND1
APC[63]	Онкосупрессор, негативный регулятор сигнального пути Wnt, способствует деградации B-катенина	Подавление экспрессии гена APC ведет к дисрегуляции в системе компонентов канонического пути Wnt/ß-катенин и усилению роста клеток опухоли
PAX1[71]	Транскрипционный фактор с предполагаемой опухоль-супрессорной активностью	Снижение экспрессии PAX1 нивелирует супрессорную активность транскрипционного фактора
СTNNB1[63]	β-катенин — ключевой компонент сигнального пути Wnt	Дисрегуляция в системе компонентов канонического пути Wnt/ß-катенин и усилению роста клеток опухоли
SFRP1, SFRP2, SFRP4[72]	Ингибиторы пути Wnt/β-катенин	Эпигенетический сайленсинг генов SFRP ведет к дисрегуляции сигнального пути Wnt/ß-катенин, связанного с развитием злокачественных опухолей
CDKN2A, CDKN2B[72]	Ингибиторы циклинзависимой киназы, негативные регуляторы роста клеток путем ингибирования клеточного цикла	Подавление экспрессии генов CDKN2A, CDKN2B ведет к пролиферации опухолевых клеток
PRDM2/RIZ1[73]	PRDM2 — белок цинкового пальца, участвует в транскрипционной регуляции	Предполагается, что сайленсинг гена PRDM2/RIZ1 устраняет его супрессорную активность
WT1[64]	WT1 — транскрипционный фактор, определяет экспрессию множества генов, участвующих в онкогенезе	Подавление экспрессии гена WT1 устраняет его супрессорную активность

## ЗАКЛЮЧЕНИЕ

В представленном обзоре рассмотрены современные аспекты классификации, морфологии образований ОЩЖ и этиологии ПГПТ, а также обобщены генетические нарушения, имеющие значение в патогенезе заболевания. Для спорадических аденом ключевыми генами, ассоциированными с их развитием, остаются MEN1 и CCND1, для карцином — CDC73 и CCND1. В отношении хромосомных аббераций при аденомах ОЩЖ наиболее часто изменения касаются 11-й хромосомы, в то время как для рака чаще выявлялись потери 1p и 13q. Выявлено и описано большое количество других потенциальных генов-кандидатов, однако их роль в онкогенезе ОЩЖ остается не до конца понятной. Можно предположить, что многообразие генов, вариантов и типов мутаций, а также различий в эпигенетических механизмах регуляции, выявляемые как при доброкачественном, так и злокачественном поражении ОЩЖ, в каждом отдельном случае создают уникальную комбинацию событий, приводящих к дестабилизации генетического аппарата клетки и ее последующей опухолевой трансформации. Доступные на сегодняшний день литературные данные ставят перед исследователями все новые вопросы, ответы на которые, возможно будут получены при появлении новых методов генетического анализа.

## ДОПОЛНИТЕЛЬНАЯ ИНФОРМАЦИЯ

Источник финансирования. Исследование выполнено за счет средств гранта РНФ. Название проекта “Геномный, транскриптомный и иммуногистохимический профиль при первично множественном поражении околощитовидных желез”. Номер проекта: 24-15-00269.

Конфликт интересов. Авторы декларируют отсутствие явных или потенциальных конфликтов интересов, связанных с публикацией настоящей статьи.

Участие авторов. Все авторы одобрили финальную версию статьи перед публикацией, выразили согласие нести ответственность за все аспекты работы, подразумевающую надлежащее изучение и решение вопросов, связанных с точностью или добросовестностью любой части работы.
